# Pathologically confirmed advanced stage of limbic predominant age related TDP‐43 encephalopathy (LATE): A case report

**DOI:** 10.1002/alz.092271

**Published:** 2025-01-03

**Authors:** Young Hee H Jung, Jin Hwan H Lee, Sung‐Hye Park

**Affiliations:** ^1^ Myongji Hospital, School of Medicine, Hanyang University, Goyang Korea, Republic of (South); ^2^ Myoungji Hospital, Goyang Korea, Republic of (South); ^3^ Seoul National University, School of Medicine, Seoul Korea, Republic of (South)

## Abstract

**Background:**

We herein introduce a case that was pathologically confirmed as an advanced stage of LATE.

**Method:**

(case presentation) An 81‐year‐old woman visited a memory clinic complaining of memory impairment in the past few years. She had no psychiatric history and a history of significant hyperlipidemia and hypertension. On MMSE she scored 20 with CDR of 1 (SOB 6). T In the first‐year post‐onset, brain MRI indicated diffuse brain atrophy with hippocampal atrophy MTA‐Gr2). At the fifth year, the patient was unable to perform all ADL and frequently exhibited severe BPSD. Contrary to the worsening of clinical symptoms, brain CT revealed only slight increase in hippocampal atrophy (MTA‐Gr3), with no significant difference from the MRI findings 5 years ago. The patient died at the age of 88.

**Result:**

The brain autopsy revealed global brain atrophy with 923g in brain weight. Final pathological diagnosis was Alzheimer’s disease (AD), limbic‐predominant age‐related TDP43 encephalopathy (LATE), and cerebrovascular disease. In detail, under the NIA‐AA ADNP grading, the level is intermediate, with scores of A3, B3, and C2 adding up to 8. The Thal phase is rated 3 out of a possible 5. The Braak stage, which indicates the progression of Alzheimer’s tau pathology, is at stages V to VI out of VI. The CERAD score was 2 out of 3. No evidence of cerebral amyloid angiopathy (CAA) was found (score of 0). The stage of LATE was 6 by Joseph staging system, thus, TDP43‐positive intraneuronal inclusions and neurites were found in the amygdala, entorhinal and temporal lobes, hippocampal cornu ammonis and dentate, basal ganglia, and tectum of the midbrain and olivary nucleus of the medulla oblongata. However, there was no hippocampal sclerosis.

**Conclusion:**

In this report, the subject was initially diagnosed with Alzheimer’s dementia while alive; however, a post‐mortem autopsy revealed the primary condition to be an advanced stage of LATE, specifically at Joseph stage 6, coexisting with Alzheimer’s disease and cerebrovascular pathology. This case underscores the importance of understanding the pathological characteristics of TDP‐43 protein abnormalities and their related clinical manifestations.

